# Care Needs of Patients With Chronic Cardiovascular Diseases: An Integrative Review

**DOI:** 10.1002/hsr2.73196

**Published:** 2026-09-09

**Authors:** Mahdi Nabi Foodani, Masoumeh Zakerimoghadam, Shahrzad Ghiyasvandian, Verónica V. Márquez Hernández, Mª Carmen Rodríguez‐García, Amir Hossein Goudarzian, Zahra Abbasi Dolatabadi

**Affiliations:** ^1^ Department of Medical Surgical Nursing School of Nursing and Midwifery Tehran University of Medical Sciences Tehran Iran; ^2^ Nursing and Midwifery Research Center Tehran University of Medical Sciences Tehran Iran; ^3^ Department of Nursing, Physiotherapy and Medicine Faculty of Health Sciences University of Almería Almería Spain; ^4^ Research Group CTS-1127 Epidemiology and Public Health University of Almería Almería Spain; ^5^ Department of Psychiatric Nursing School of Nursing and Midwifery Mazandaran University of Medical Sciences Sari Iran

**Keywords:** cardiovascular disease, chronic illness, nurse, nursing care

## Abstract

**Background and Aims:**

Chronic cardiovascular diseases (CVDs) remain a primary contributor to global mortality. Despite their prevalence, the comprehensive care needs of affected patients are not fully elucidated. This integrative review sought to synthesize current evidence on the multifaceted care requirements of individuals with chronic CVDs.

**Methods:**

A comprehensive literature search was performed using PubMed, Scopus, and Web of Science, targeting studies published between 2010 and February 2025. The Whittemore and Knafl framework guided the methodology, incorporating both quantitative and qualitative research. Thematic analysis was utilized to distill key care‐related themes.

**Results:**

The analysis of 14 studies revealed 5 predominant themes central to the care needs of patients with chronic CVDs. Health Education encompassed the dissemination of medical information, lifestyle education, guidance on using medical instruments, promotion of healthy behaviors, and counseling on diet and nutrition. Self‐Care Management focused on self‐care practices, medication adherence, symptom monitoring, risk factor control, and strategies to prevent complications. Holistic Support included mental health resources, emotional reassurance, support groups, assistance with daily activities, engagement of family and community, caregiver support, cost management, and socioeconomic aid. Care Coordination highlighted the importance of primary contact persons, telehealth and digital health solutions, and accessibility to healthcare services. Lastly, End‐of‐Life Care addressed decision‐making processes, palliative care consultations, and attention to spiritual and existential needs.

**Conclusion:**

This review underscores the complex and diverse care needs of patients with chronic CVDs, advocating for the adoption of comprehensive, patient‐centered care models. These insights provide a foundation for designing targeted interventions and directing future research to enhance patient outcomes and quality of life.

## 1 Introduction

Chronic cardiovascular diseases (CVDs) are among the most significant health challenges worldwide, imposing a substantial burden on public health and healthcare systems [1]. According to the World Health Organization, these diseases account for approximately 17.9 million deaths annually, representing 31% of all global deaths. The prevalence of these diseases is steadily increasing due to aging populations, the rise of risk factors such as hypertension, diabetes, obesity, and unhealthy lifestyles [2]. Beyond their extensive physical impacts, the consequences of these conditions include psychological disorders and reduced quality of life, which have long‐term effects on patients and their families [3]. Addressing these challenges is critical, as unmet care needs not only exacerbate patient outcomes but also strain healthcare systems, leading to increased costs and reduced efficiency in care delivery.

Previous studies have shown that patients with CVDs have diverse needs that extend beyond the management of physical symptoms. For instance, research by Peng et al. [4] and Borkowski and Borkowska [5] highlights that these patients face psychological challenges such as anxiety and depression, social difficulties like insufficient family support, and a need for better information on self‐management. Additionally, a systematic review by Doherty et al. [6] revealed that existing interventions primarily focus on medical treatments, while the emotional and social needs of patients are not adequately addressed. Although these studies provide valuable insights, there is a noticeable lack of a comprehensive perspective that examines the multifaceted needs of patients across diverse cultural and social contexts.

This knowledge gap indicates that, despite the importance of nonmedical needs, there is still insufficient research to fully explain and integrate these needs. Many existing care approaches have limited effectiveness due to an incomplete understanding of patients' needs, potentially leading to reduced patient satisfaction and quality of life [7]. Furthermore, rapid changes in lifestyle patterns and social structures, particularly in developing societies, have heightened the need to update existing knowledge in this field [8].

Therefore, the present study aims to provide a comprehensive understanding of the care needs of patients with CVDs in order to address the previous knowledge gaps. To achieve this, an integrative review has been conducted, utilizing both quantitative and qualitative research articles to provide with a more holistic and multidimensional perspective. This method, by synthesizing diverse findings from various sources, enables the identification of less‐explored aspects and the development of more practical, evidence‐based solutions. Additionally, the integrative review helps analyze current trends and knowledge gaps, providing a strong foundation for designing effective interventions tailored to the needs of patients.

## 2 Methods

### 2.1 Study Design

This study is designed as an integrative review based on the framework proposed by Whittemore and Knafl [9]. The aim of this type of review is to synthesize findings from various studies (quantitative, qualitative, and mixed methods) to provide a comprehensive and multidimensional understanding of the topic under investigation. The main stages of this review include problem identification, comprehensive search, quality assessment, data analysis, and presentation of results. The research problem was defined as follows: “What are the care needs of patients with chronic cardiovascular diseases?” The question was formulated using the SPIDER framework to explicitly define the scope of the evidence and guide the eligibility criteria and search strategy [10].

The SPIDER components were operationalized as follows: Sample (S): Individuals of any age diagnosed with or living with chronic CVDs. Chronic CVDs were operationally defined as cardiovascular conditions requiring ongoing or long‐term management, follow‐up, or care because of their persistent or recurrent nature, including chronic heart disease, heart failure (HF), hypertension, dyslipidemia, valvular heart disease, chronic vascular conditions, and other cardiovascular conditions explicitly described as chronic by the primary study; Phenomenon of Interest (PI): patients' care needs, including physical, psychological, social, educational, self‐care, supportive, care‐coordination, and end‐of‐life needs; Design (D): quantitative, qualitative, and mixed‐methods empirical study designs; Evaluation (E): reported or explored care needs, experiences, perceptions, priorities, preferences, challenges, or unmet needs related to living with chronic CVDs; and Research type (R): qualitative, quantitative, and mixed‐methods research.

Accordingly, studies were eligible when they included individuals with chronic CVDs (S), addressed care needs or related experiences and priorities (PI/E), and used an eligible empirical research design (D/R). The SPIDER framework therefore served as the conceptual basis for defining the population and phenomenon of interest, identifying the types of outcomes or experiences sought, and determining eligible study designs and research types.

For the purpose of this review, chronic CVDs were operationally defined as cardiovascular conditions that require ongoing or long‐term management, follow‐up, or care because of their persistent or recurrent nature. This included chronic heart disease, HF, hypertension, dyslipidemia, valvular heart disease, chronic vascular conditions, and other cardiovascular conditions when the study explicitly addressed their chronic or long‐term care needs. Acute cardiovascular conditions without a chronic disease component were not considered eligible.

### 2.2 Search Strategy

A comprehensive search was conducted in PubMed, Scopus, Web of Science, ProQuest, Google Scholar, and Persian databases including SID and Magiran. The search strategy was developed from the SPIDER framework. The Sample (S) and Phenomenon of Interest (PI) components primarily informed the electronic search terms. Accordingly, combinations of controlled vocabulary (e.g., MeSH terms) and free‐text terms representing chronic CVDs and care‐related needs were developed using the Boolean operators AND and OR. The Evaluation (E) component was represented by terms related to patients' needs and care experiences, including “care needs,” “patient needs,” and “nursing care.” Because Design (D) and Research type (R) are methodological rather than population or phenomenon concepts, they were primarily applied during the screening and eligibility assessment rather than being used as restrictive database search terms.

This approach was adopted to maximize the sensitivity of the search and avoid excluding relevant qualitative, quantitative, or mixed‐methods studies because of differences in methodological terminology or indexing. Reference lists of included studies and gray literature, including theses and organizational reports, were also reviewed to identify potentially relevant evidence. The initial searches were conducted by the first author and a professional librarian at the University in June 2024 and were updated in February 2025 to identify newly published or indexed studies. The final search therefore included evidence available up to February 2025. The database‐specific search strategies are reported in Table 1.

**Table 1:** Search strategy.

Database	Search strategy	Results
PubMed	“Cardiovascular diseases”[MeSH Terms] OR “chronic cardiovascular disease”[Title/Abstract] OR “chronic heart disease”[Title/Abstract] OR “heart failure”[Title/Abstract] OR “hypertension”[Title/Abstract] OR “varicose”[Title/Abstract] OR “dyslipidemia”[Title/Abstract] OR “heart valve disease”[Title/Abstract] AND “nursing care”[MeSH Terms] OR “care needs”[Title/Abstract] OR “patient needs”[Title/Abstract]	644
Scopus	(TITLE‐ABS‐KEY (“Chronic Cardiovascular Disease” OR “Chronic Heart Disease” OR “chronic heart disease” OR “heart failure” OR “hypertension” OR “varicose” OR “dyslipidemia” OR “heart valve disease” AND TITLE‐ABS‐KEY (“nursing care” OR “care needs” OR “patient needs”))	2163
Web of Science	TS= (“Chronic Cardiovascular Disease” OR “Chronic Heart Disease” OR “chronic heart disease” OR “heart failure” OR “hypertension” OR “varicose” OR “dyslipidemia” OR “heart valve disease”) AND TS= (“nursing care” OR “care needs” OR “patient needs”)	1258
ProQuest	(“Chronic Cardiovascular Disease” OR “Chronic Heart Disease” OR “chronic heart disease” OR “heart failure” OR “hypertension” OR “varicose” OR “dyslipidemia” OR “heart valve disease”) AND (“nursing care” OR “care needs” OR “patient needs”)	0
SID	(“Chronic Cardiovascular Disease” OR “Chronic Heart Disease” OR “chronic heart disease” OR “heart failure” OR “hypertension” OR “varicose” OR “dyslipidemia” OR “heart valve disease”) AND (“nursing care” OR “care needs” OR “patient needs”)	1
Magiran	(“Chronic Cardiovascular Disease” OR “Chronic Heart Disease” OR “chronic heart disease” OR “heart failure” OR “hypertension” OR “varicose” OR “dyslipidemia” OR “heart valve disease”) AND (“nursing care” OR “care needs” OR “patient needs”)	0
Total	—	4066

### 2.3 Screening

The retrieved studies underwent a two‐stage screening process. In the first stage, the titles and abstracts of the articles were independently reviewed by two researchers, and irrelevant studies were excluded. In the second stage, the full texts of the articles were examined, and only studies that met the inclusion criteria were included in the analysis. Any disagreements between the researchers were resolved through discussion or by consulting a third reviewer. The inclusion criteria were derived from the SPIDER framework. Studies were eligible if they: (1) included individuals with chronic CVDs, irrespective of age (S); (2) investigated or reported patients' care needs or related experiences, perceptions, priorities, preferences, challenges, or unmet needs (PI/E); and (3) used a qualitative, quantitative, or mixed‐methods empirical design (D/R). Studies published between January 2010 and February 2025 were eligible for inclusion.

The population was not restricted by age; therefore, studies involving pediatric, adolescent, adult, or older adult patients with chronic CVDs were eligible. Pediatric populations were specifically considered eligible when participants met the operational definition of chronic CVD and the study addressed their care needs.

The exclusion criteria included studies unrelated to the review question, letters to the editor, commentaries, review studies, studies that did not meet the predefined methodological quality threshold, and studies for which the full text was unavailable. Studies exclusively involving acute cardiovascular conditions without an underlying chronic cardiovascular condition were excluded.

**Figure 1:**
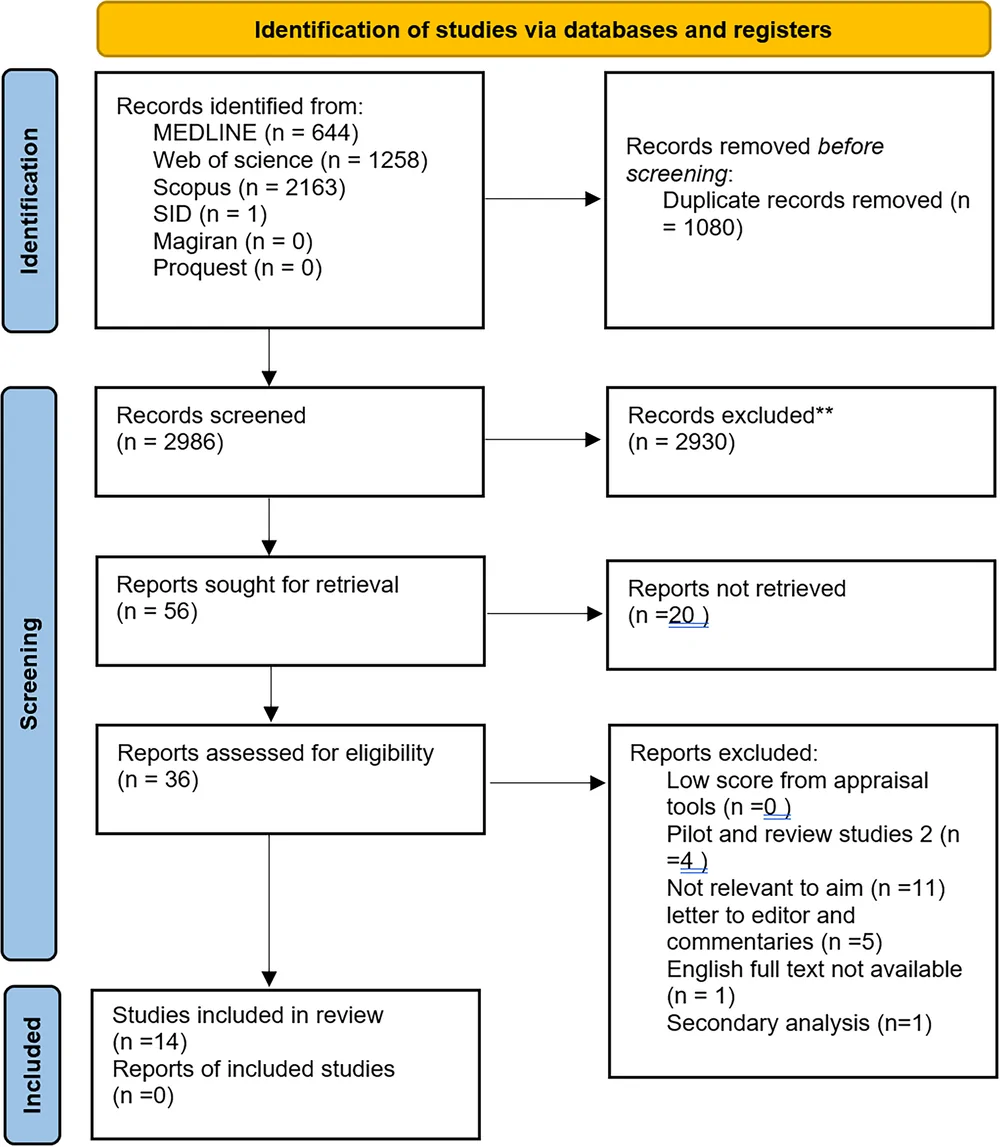
PRISMA flowchart.

Key information was extracted from the selected studies, including article details (authors, year of publication, country of study), methodology (study type, population, data collection tools), and main findings. The process of study selection is illustrated in the PRISMA flow diagram (Figure 1). The diagram outlines the total number of records identified, duplicates removed, and the number of studies excluded at each stage of the screening process.

While our comprehensive initial search yielded 4066 records, our inclusion criteria were specifically designed to prioritize empirical research that directly addressed the multifaceted care needs of patients with chronic CVDs. The reduction from initial records to final inclusion reflects the exclusion of studies that did not fit with the aim of this study and did not meet our threshold for methodological quality, those that were not relevant to the specific research aim, and the removal of duplicate records, commentaries, and secondary analyses.

At the full‐text assessment stage, 36 reports were assessed for eligibility. A total of 22 reports were excluded, with specific reasons recorded: 11 studies were not relevant to the research aim, 5 were letters to the editor or commentaries, 4 were pilot or review studies rather than original research, 1 had no full‐text available in English, and 1 was a secondary analysis.

### 2.4 Quality Assessment

The methodological quality of the included studies was independently assessed by two reviewers using the Joanna Briggs Institute (JBI) critical appraisal tools appropriate to each study design [11, 12] (Tables 2 and 3). The 7 qualitative studies were evaluated using the JBI Critical Appraisal Checklist for Qualitative Research, which comprises 10 items assessing congruity between the stated philosophical perspective, methodology, research question, data collection, analysis, and interpretation, as well as researcher reflexivity, representation of participants' voices, ethical conduct, and the extent to which conclusions were grounded in the analysis.

**Table 2:** JBI Qualitative Research Checklist.

Study	Q1	Q2	Q3	Q4	Q5	Q6	Q7	Q8	Q9	Q10	Yes/10	Rating
Blair et al. [13]	U	Y	Y	Y	Y	N	N	Y	U	Y	6/10	Moderate
Baudendistel et al. [14]	U	Y	Y	Y	Y	N	N	Y	U	Y	6/10	Moderate
Kimani et al. [15]	U	Y	Y	Y	Y	N	N	Y	Y	Y	7/10	Moderate
Tulloch et al. [16]	U	Y	Y	Y	Y	N	N	Y	U	Y	6/10	Moderate
Kókai et al. [17]	N	U	Y	U	Y	N	Y	U	Y	Y	5/10	Moderate
Engelmann et al. [18]	U	Y	Y	Y	Y	N	U	Y	Y	Y	7/10	Moderate
Khasanah et al. [19]	U	Y	Y	Y	Y	N	U	Y	U	Y	6/10	Moderate

**Table 3:** JBI Analytical Cross‐Sectional Studies Checklist.

Study	Q1	Q2	Q3	Q4	Q5	Q6	Q7	Q8	Yes/8	Rating
Min et al. [20]	Y	Y	Y	Y	N	N	Y	Y	6/8	Moderate
Schäfer‐Keller et al. [21]	Y	Y	Y	Y	U	N	Y	Y	6/8	Moderate
Matsunuma et al. [22]	Y	Y	Y	Y	N	N	Y	Y	6/8	Moderate
DeGroot et al. [23]	Y	Y	Y	Y	U	U	Y	Y	6/8	Moderate
Rittirong et al. [24]	Y	Y	U	U	N	Y	U	Y	4/8	Moderate
Aldawsari et al. [25]	Y	Y	U	U	N	Y	U	Y	4/8	Moderate
Schmitz et al. [26]	Y	Y	Y	Y	N	N	Y	Y	6/8	Moderate

The seven quantitative studies, all cross‐sectional in design, were evaluated using the JBI Critical Appraisal Checklist for Analytical Cross‐Sectional Studies, comprising 8 items assessing the clarity of inclusion criteria, description of subjects and setting, validity and reliability of exposure and outcome measurement, use of objective diagnostic criteria, identification of and strategies to address confounding factors, and appropriateness of statistical analysis.

Each item was rated as “Yes,” “No,” or “Unclear,” and an overall quality score was calculated as the proportion of “Yes” responses out of the total items (10 for qualitative studies, 8 for quantitative studies). Studies were categorized as high (≥ 80% of items met), moderate (50%–79%), or low (< 50%) quality. Disagreements between reviewers were resolved through discussion until consensus was reached. Only studies rated as moderate to high quality were retained for inclusion in the review.

### 2.5 Data Analysis

Data were analyzed using a structured thematic synthesis approach consistent with the integrative review methodology described by Whittemore and Knafl. Because the included evidence comprised both quantitative and qualitative studies, findings from the two evidence types were first extracted and examined separately and were subsequently integrated at the level of care needs.

For quantitative studies, findings relevant to patients' care needs were extracted from the results, including reported frequencies, proportions, rankings, mean scores, and other quantitative indicators of the importance or prevalence of specific needs. These findings were converted into descriptive statements representing the underlying care need rather than statistically pooled, because the included studies differed in populations, outcomes, measurement instruments, and study contexts. For qualitative studies, findings relevant to patients' care needs were extracted from reported themes, categories, subcategories, and participants' experiences or perceptions. These findings were summarized using descriptive codes representing the content of the identified care needs.

The findings from both evidence types were then compared and integrated using a convergent thematic synthesis approach. First, findings with similar meanings or content were grouped into preliminary codes. Second, related codes were compared across studies and across quantitative and qualitative evidence and were clustered into descriptive subthemes. Third, conceptually related subthemes were reviewed and grouped into broader analytical themes. During this process, similarities and differences between quantitative and qualitative findings were examined, and findings that addressed the same underlying care need were integrated even when they were expressed using different terminology or methodological approaches.

To facilitate integration and verification, the extracted findings were organized according to study characteristics, methodological approach, and identified care needs. The distribution of findings across studies was then compared to determine whether a theme was consistently supported across different studies and evidence types or whether it was specific to particular populations or contexts.

Finally, the study results were reported in accordance with the PRISMA (2020) guidelines [27]. This stage included a flow diagram of the study selection process, summary tables of study characteristics (Table 4) and main findings, and a detailed analysis of the identified themes.

**Table 4:** Characteristics of the articles included in the study.

	First author (year)	Title of study	Main results
1	Blair et al. (2014) [13]	Challenges, needs, and experiences of recently hospitalized cardiac patients and their informal caregivers	The study found that both cardiac patients and their informal caregivers identified diet as the most pressing challenge.
2	Baudendistel et al. (2015) [14]	Bridging the gap between patient needs and quality indicators: a qualitative study with chronic heart failure patients	The study found that chronic heart failure (CHF) patients value strong doctor–patient relationships, clear communication, and individualized care, but they identified gaps in coordination and communication across healthcare sectors, especially during transitions between hospital and outpatient care.
3	Kimani et al. (2018) [15]	Multidimensional needs of patients living and dying with heart failure in Kenya: a serial interview study	The study explored the experiences of patients living with advanced heart failure in Kenya, revealing significant physical, psychosocial, spiritual, and financial distress.
4	Tulloch et al. (2020) [16]	Learning a new way of living together: a qualitative study exploring the relationship changes and intervention needs of patients with cardiovascular disease and their partners	Patients and partners expressed a need for practical resources, peer‐sharing opportunities, and relationship enhancement to better cope with CVD.
5	Min et al. (2020) [20]	Comparison of learning needs priorities between healthcare providers and patients with heart failure	The findings highlight the need for patient‐centered education that addresses the actual learning needs of HF patients while considering the perspectives of healthcare providers.
6	Schäfer‐Kelleret al. (2021) [21]	Self‐care, symptom experience, needs, and past health‐care utilization in individuals with heart failure: results of a cross‐sectional study	The study found that self‐care levels among individuals with heart failure were suboptimal, with only 24%, 10%, and 61% reporting adequate self‐care maintenance, management, and confidence, respectively.
7	Matsunuma et al. (2022) [22]	Comprehensive palliative care needs in outpatients with chronic heart failure: a Japanese cross‐sectional study	The study found that nearly half of advanced‐stage patients reported family anxiety, highlighting the need for comprehensive palliative care addressing both patient and family needs.
8	Kókai et al. (2022) [17]	Needs and preferences of women with prior severe preeclampsia regarding app‐based cardiovascular health promotion	The study found that women with prior severe preeclampsia primarily needed health promotion targeting fat and sugar intake and physical activity, with some also expressing needs related to mental health practices, fruit and vegetable intake, salt intake, and water intake.
9	DeGroot et al. (2023) [23]	Palliative care needs of physically frail community‐dwelling older adults with heart failure	The study found that physically frail older adults with heart failure experience significantly higher unmet palliative care needs compared to nonfrail individuals.
10	Rittirong et al. (2023) [24]	Patients' need for sexual counseling in the cardiac rehabilitation service	Most patients (91.2%) agreed that healthcare providers should proactively offer sexual counseling, preferably through direct consultation.
11	Engelmann et al. (2023) [18]	Needs of multimorbid heart failure patients and their carers: a qualitative interview study and the creation of personas as a basis for a blended collaborative care intervention	The study identified three personas representing the needs of multimorbid heart failure patients and their carers: (1) "The one who needs and wants support," (2) "The one who has accepted their situation with heart failure and reaches out, when necessary," and (3) "The one who feels neglected by the health care system." These personas highlight the diverse needs for education, emotional support, and personalized care, with carers of the third persona experiencing high psychological stress.
12	Aldawsari et al. (2024) [25]	Assessing the health education needs of heart failure patients in Saudi Arabia	Patients strongly preferred doctors as educators (79.5%) and rated medical information, lifestyle modifications, and complication prevention as their top health education needs.
13	Schmitz et al. (2024) [26]	Living lab data of patient needs and expectations for eHealth‐based cardiac rehabilitation in Germany and Spain from the TIMELY study: cross‐sectional analysis	Patients expressed a high need for personalized, user‐friendly eHealth platforms that support regular physical exercise, provide updates on training recommendations, and offer access to medical information like diagnoses and test results.
14	Khasanah et al. (2024) [19]	The problems and needs of self‐management among Indonesian older adults with hypertension: a qualitative study	The study identified eight main themes related to the problems and needs of self‐management among Indonesian older adults with hypertension, including complaints related to aging and hypertension, inadequate knowledge about hypertension, difficulty in changing behavior, lack of family and health service support, and noncompliance with medication.

### 2.6 Ethics Approval

This integrative review compiles data from previously published studies, without the direct involvement of new human or animal subjects or the collection of primary data. Ethical approval and informed consent were secured in the original studies included in this review. The research was conducted following ethical guidelines for reviews, maintaining transparency, accuracy, and compliance with established reporting standards such as PRISMA.

## 3 Results

In total, 14 studies were included in the review [20, 21, 22, 23, 24, 25, 26] comprising 7 quantitative and 7 qualitative studies (Table 4) [13, 14, 15, 16, 17, 18, 19]. Findings from both evidence types were first examined separately and subsequently integrated through thematic synthesis. Similar findings across studies were grouped into preliminary codes, which were then compared and clustered into subthemes. Related subthemes were further synthesized into five overarching themes: Health Education, Self‐Care Management, Holistic Support, Care Coordination, and End‐of‐Life Care. The final themes were reviewed against the extracted findings to ensure that they adequately represented the evidence across the included studies (Table 5).

**Table 5:** Main themes and subthemes generated through the integrative thematic synthesis.

Main themes	Subthemes
Health Education	Medical Information About the Disease
	Lifestyle Education
	Use of Medical Instruments
	Healthy Behaviors
	Diet and Nutrition
Self‐Care Management	Self‐Care Behaviors
	Medication Management
	Symptom Monitoring
	Risk Factor Management
	Prevention of Complications
Holistic Support	Mental Health Support
	Reassurance
	Support Groups
	Daily Activity Assistance
	Family Involvement
	Community Support
	Caregiver Support
	Cost Management
	Socioeconomic Assistance
Care Coordination	Primary Contact Person
	Telehealth and Digital Health Tools
	Access to Healthcare Services
End‐of‐Life Care	End‐of‐Life Decision‐Making
	Palliative Care Consultation
	Spiritual and Existential Needs

Across the five themes, the number and methodological composition of contributing studies varied. Health Education was supported by six studies (three qualitative and three quantitative) and showed high consistency across study designs and settings. Self‐Care Management was identified in four studies (two qualitative and two quantitative), with consistent evidence for the importance of self‐care support. Holistic Support was supported by five studies (three qualitative and two quantitative), although some divergence was observed between qualitative reports of patients' support needs and quantitative associations with health behaviors.

Care Coordination was identified in three studies (one qualitative and two quantitative) and showed lower and more context‐dependent consistency, particularly because evidence was concentrated in higher‐income settings. End‐of‐Life Care was supported by four studies (three qualitative and one quantitative); the direction of findings was generally consistent, although the magnitude of reported unmet needs varied across patient groups. Because individual studies could contribute to more than one theme, the numbers of studies across themes are not additive. Detailed information on the contributing study types, consistency, and areas of heterogeneity is provided in Table 6.

**Table 6:** Number, design type, consistency, and contextual heterogeneity of evidence contributing to each theme.

Theme	Studies (*n*)	Design mix	Consistency	Key tension/contradiction
Health Education	6	3 QUAL, 3 QUAN	High (consistent across designs and settings)	QUAN studies prioritize content (disease/medication knowledge); QUAL studies prioritize communication style
Self‐Care Management	4	2 QUAL, 2 QUAN	High (need consistently reported)	Recognized need for support, but Schäfer‐Keller found actual self‐care adequacy remains low
Holistic Support	5	3 QUAL, 2 QUAN	Moderate (qualitative and quantitative evidence diverge)	Nordin and colleagues found weak statistical link between emotional support and lifestyle change, despite strong qualitative demand
Care Coordination	3	1 QUAL, 2 QUAN	Low (moderate context‐dependent)	Emphasized mainly in higher‐income settings (Germany, Spain, Canada); largely absent from LMIC studies
End‐of‐Life Care	4	3 QUAL, 1 QUAN	Moderate (direction consistent, magnitude varies)	DeGroot and colleagues: frail patients report substantially higher unmet need than nonfrail patients with the same diagnosis

### 3.1 Health Education

Health Education was supported by six studies, comprising three quantitative and three qualitative studies, and demonstrated high consistency across study designs and healthcare settings [13, 25, 26]. Quantitative surveys primarily focused on educational priorities such as disease knowledge, medication use, and lifestyle modification, whereas qualitative studies highlighted patients' desire for individualized communication, understandable information, and opportunities to ask questions. Collectively, these findings suggest that effective education extends beyond information provision and requires patient‐centered communication tailored to individual learning needs.

Patients require education about the nature of their illness, how to manage symptoms, and how to prevent complications. This includes understanding medical terminology, using medical devices, and the importance of avoiding unhealthy behaviors such as smoking or excessive alcohol consumption. Effective health education empowers patients to take an active role in managing their condition. Subthemes of this main theme include: Medical Information About the Disease, Lifestyle Education, Use of Medical Instruments, Healthy Behaviors, and Diet and Nutrition.

#### 3.1.1 Medical Information About the Disease

Patients need comprehensive medical information about their disease, including its nature, progression, and management, to better understand their condition. This includes understanding the cause of their illness, the diagnostic process, and their prognosis, as well as explanations of test results and the role of medications in their treatment [20, 28].

#### 3.1.2 Lifestyle Education

Lifestyle education is equally important, as patients benefit from guidance on adopting healthy behaviors, such as reducing salt intake, increasing physical activity, and managing stress effectively. They should also be educated on how specific lifestyle changes, like improving their diet and incorporating regular exercise, can significantly reduce their risk of CVD [15, 21, 24].

#### 3.1.3 Use of Medical Instruments

Training on the use of medical instruments is a crucial component of patient education, particularly for those managing chronic conditions like CVD. Patients may need to learn how to operate devices such as blood pressure monitors to regularly track their blood pressure at home, ensuring they can monitor their health and detect any concerning changes early [16].

#### 3.1.4 Healthy Behaviors

Healthy behaviors are critical for patients with CVD, and they require guidance on safe physical activities and exercise programs tailored to their specific condition [17].

#### 3.1.5 Diet and Nutrition

Diet and nutrition play a vital role in managing CVD, and patients need practical advice on making dietary changes to support their heart health. This includes reducing salt intake, following a heart‐healthy diet, and adhering to cardiac dietary guidelines. Patients may also benefit from guidance on meal preparation to ensure they can maintain these dietary changes in their daily lives [15, 26].

### 3.2 Self‐Care Management

Self‐Care Management was supported by four studies, including two quantitative and two qualitative studies. Findings were highly consistent in identifying self‐care support as an important patient need. Self‐care management focuses on the daily practices patients must adopt to manage their condition effectively. This includes monitoring symptoms, adhering to medication regimens, and making lifestyle changes such as dietary adjustments and regular physical activity. Caregivers also play a critical role in supporting patients, especially when they struggle with self‐care tasks. This theme highlights the importance of providing patients with the tools and knowledge to manage their condition independently. The subthemes of this main theme are the following: Self‐Care Behaviors, Medication Management, Symptom Monitoring, Risk Factor Management, and Prevention of Complications [13, 14, 18, 21].

#### 3.2.1 Self‐Care Behaviors

Self‐care behaviors are essential for patients managing chronic conditions like HF. Patients require support in performing basic self‐care tasks, such as monitoring symptoms and adhering to medication regimens, to maintain their health and prevent complications [16]. This includes regular weight monitoring, fluid restriction, and strict dietary adherence to manage their condition effectively. Additionally, patients need to be educated on recognizing key symptoms of HF, such as dyspnea and edema, and taking appropriate countermeasures, like reducing salt intake, regulating fluid intake, or taking extra diuretics as prescribed [21].

#### 3.2.2 Medication Management

Medication management is another critical aspect of self‐care, as patients often need assistance with taking prescribed medications correctly. This involves understanding proper dosages, potential side effects, and the importance of adherence to their treatment plan. Patients should also be educated on managing medication side effects and avoiding potential drug interactions. Developing a system, such as pill organizers or reminders, can help patients remember to take their medications consistently and on time [15].

#### 3.2.3 Symptom Monitoring

Symptom monitoring is a key skill for patients, as it enables them to track their health status and respond to changes promptly. Patients should be taught how to monitor symptoms like dyspnea and edema and recognize when to seek medical help. They also need guidance on lifestyle adjustments, such as reducing salt intake, regulating fluid intake, and knowing when to take additional diuretics to manage fluid retention [18, 22].

#### 3.2.4 Risk Factor Management

Risk factor management is a cornerstone of preventing further cardiovascular events in patients with CVD. Education on managing key risk factors, such as hypertension, diabetes, and high cholesterol, is essential to help patients take control of their health [13, 21].

#### 3.2.5 Prevention of Complications

Preventing complications is another key focus, with education on how to avoid serious issues related to CVD, such as HF. This involves managing risk factors like hypertension, diabetes, and high cholesterol through lifestyle changes and medical interventions. Patients may also need training on how to use medical instruments, such as blood pressure monitors or pacemakers, to monitor and manage their condition effectively [19, 25].

### 3.3 Holistic Support

Holistic Support was identified in five studies, comprising three qualitative and two quantitative studies. The overall evidence was moderate and showed greater heterogeneity than the Health Education and Self‐Care Management themes. Qualitative studies emphasized patients' needs for emotional, psychological, social, family, and community support, whereas quantitative evidence was more likely to examine relationships between support and health‐related behaviors or outcomes. For example, one quantitative study found only a weak association between emotional support and adoption of healthy lifestyles, despite qualitative evidence indicating a strong perceived need for emotional and social support. This difference suggests that the experience or perceived need for support does not necessarily translate into a measurable association with behavioral outcomes.

#### 3.3.1 Mental Health Support

Mental health support is a critical aspect of care for patients with CVD, as they often experience anxiety, depression, and emotional distress due to the challenges of living with a chronic condition [15, 17].

#### 3.3.2 Reassurance

Reassurance plays a vital role in helping patients feel more secure about their health status, particularly after hospitalizations or when they feel uncertain about their symptoms. Emotional support is equally important for patients and their partners, especially when coping with changes in sexual health, which can be a sensitive and distressing aspect of their condition [23, 24].

#### 3.3.3 Support Groups

Support groups offer a valuable resource for both patients and caregivers, as they provide a space to share experiences, coping strategies, and emotional support. Patients expressed a desire for peer support to normalize their feelings and learn from the successes of others who face similar challenges. These groups can foster a sense of community and reduce feelings of isolation, helping patients and caregivers navigate the complexities of living with CVD [14, 16].

#### 3.3.4 Daily Activity Assistance

Daily activity assistance may be necessary for some patients, particularly those with physical limitations caused by their condition. This can include help with housework, exercises, or leisure activities to ensure they can maintain independence and enjoy a fulfilling lifestyle despite their health challenges [15, 23].

#### 3.3.5 Community Support

Community support is another valuable resource for CVD patients, as community‐based programs, such as support groups or health education sessions, can provide practical tools and emotional encouragement [13, 15].

#### 3.3.6 Family Involvement

Family involvement plays a crucial role in the care of CVD patients, as they often rely on informal caregivers, such as family members, partners, or friends, for support. However, caregivers themselves may need guidance and assistance in managing their responsibilities [13, 16].

#### 3.3.7 Caregiver Support

Caregiver support plays a vital role in promoting self‐care behaviors, especially when patients face challenges with adherence or symptom management. Caregivers, often family members, need education on how to support the patient's self‐care routines, including medication management, dietary adherence, and symptom monitoring. By working together, patients and caregivers can create a supportive environment that enhances the patient's ability to manage their condition and improve their quality of life [13, 14, 18].

#### 3.3.8 Cost Management

Cost management is a significant concern for patients with CVD, as they often face a substantial financial burden due to the cost of medications, treatments, and healthcare services. Many patients struggle with financial challenges related to medical bills and insurance coverage, which can create stress and hinder their ability to adhere to treatment plans. Addressing these financial barriers is essential to ensure patients can access the care they need without compromising their financial stability [13, 20].

#### 3.3.9 Socioeconomic Assistance

Socioeconomic assistance is also critical for many patients, as they may face sociolegal issues related to accessing services for people with CVD and comorbidities or navigating the complexities of the healthcare system. Addressing financial barriers, such as the cost of medications and transportation, can help ensure that patients receive the care they need [19, 22].

### 3.4 Care Coordination

Care Coordination was supported by three studies, including one qualitative and two quantitative studies, representing the least extensive evidence base among the five themes. The evidence was rated as low to moderate in consistency and was strongly context‐dependent. The need for coordinated care, continuous communication, and access to services was identified in the contributing studies; however, these needs were emphasized primarily in higher‐income settings, including Germany, Spain, and Canada. In contrast, studies conducted in low‐ and middle‐income settings did not identify named care coordinators or telehealth platforms as prominent priorities and instead emphasized basic access to healthcare services and affordability.

#### 3.4.1 Primary Contact Person

Having a primary contact person, often their general practitioner, is highly valued by patients, as this individual serves as a central point of communication and coordinates their care across various healthcare providers. This ensures that patients receive consistent guidance and support, particularly regarding self‐care and symptom management, which are critical for managing their condition effectively [13].

#### 3.4.2 Telehealth and Digital Health Tools

Telehealth and digital health tools are increasingly important in modern healthcare, offering innovative ways to support patients. Utilizing digital tools, such as mobile apps or online platforms, can provide education and support for self‐management, empowering patients to take an active role in their care. Remote consultations and follow‐ups through telehealth can also improve access to care, particularly for patients in remote or underserved areas, ensuring they receive the support they need without the barriers of distance or travel [17, 26].

#### 3.4.3 Access to Healthcare Services

Access to healthcare services is another key component of care coordination. Patients need timely access to medical care, especially during acute health crises, to prevent complications and ensure prompt treatment. Additionally, the availability of specialized services, such as cardiac rehabilitation programs, exercise groups, and self‐help groups, can provide patients with the resources they need to manage their condition and improve their quality of life [15, 22].

### 3.5 End‐of‐Life Care

End‐of‐Life Care was supported by four studies, comprising three qualitative and one quantitative study. The direction of findings was generally consistent, providing moderate evidence for the importance of palliative care, end‐of‐life decision‐making, and spiritual or existential support; however, the magnitude of unmet needs varied across patient groups. In particular, physically frail community‐dwelling older adults with HF reported substantially greater unmet palliative care needs than nonfrail patients with the same diagnosis, suggesting that frailty and disease trajectory may influence the intensity of end‐of‐life care needs.

#### 3.5.1 End‐of‐Life Decision‐Making

End‐of‐life decision‐making is a critical and sensitive aspect of care for patients with advanced CVD, particularly those who are physically frail. These patients often face complex decisions regarding their treatment preferences and end‐of‐life care, requiring open and compassionate discussions to ensure their wishes are respected. Engaging patients and their families in these conversations helps align care with their values and goals, providing clarity and peace of mind during a challenging time [22, 23].

#### 3.5.2 Palliative Care Consultation

Palliative care consultation plays a vital role in supporting patients with advanced CVD. Integrating palliative care early in the disease trajectory can help address the symptom burden, such as pain, fatigue, and breathlessness, while improving the patient's overall quality of life. As patients approach the end of life, palliative care focuses on providing comfort, managing symptoms, and ensuring dignity, allowing them to live as fully and comfortably as possible [22, 23].

#### 3.5.3 Spiritual and Existential Needs

Addressing spiritual and existential needs is equally important for patients facing the end of life. Many experience spiritual or existential distress, including fears of death and dying, and may struggle to find meaning in their illness. Providing spiritual support, whether through chaplaincy services, counseling, or other resources, can help patients navigate these profound concerns and find solace [22, 23].

Overall, the five themes showed different levels of evidentiary consistency. Health Education and Self‐Care Management demonstrated the greatest convergence across quantitative and qualitative evidence, whereas Holistic Support showed some methodological divergence, and Care Coordination was particularly context‐dependent. End‐of‐Life Care showed generally consistent direction of findings but variation in the magnitude of unmet needs according to patient characteristics such as frailty. This variation was considered during synthesis rather than treated as evidence against the existence of the broader themes.

## 4 Discussion

Based on the results of the present study, the care needs of patients with CVD are categorized into five main themes: Health Education, Self‐Care Management, Holistic Support, Care Coordination, and End‐of‐Life Care. These themes encompass the diverse and multifaceted needs of patients, highlighting the importance of addressing not only their physical health but also their psychological, social, and emotional well‐being, as well as the coordination of care and support for advanced stages of the disease.

Beyond confirming that these five domains of need exist each already well established in the broader chronic‐illness literature the contribution of this review lies in showing how the weight given to each domain shifts with health‐system context, disease trajectory, and culture, and in identifying which needs remain persistently unmet despite being widely recognized in guidelines. These cross‐cutting patterns, detailed within each theme below, summarized by strength of evidence in Table 6, and expanded on later in this section, distinguish care needs that appear universal across the included studies from those that are contingent on setting and population.

Based on the results of the present study, one of the most important needs of patients with chronic CVDs is health education. This need encompasses a wide range of information, including details about the disease and treatment process, patient choices, lifestyle modifications, the use of medical devices, dietary precautions, and health behaviors. According to Mackey et al. [28], health literacy is one of the most critical needs for patients with chronic diseases, as it can significantly influence health behaviors. Although this study focuses broadly on chronic diseases, its findings align with the current study. Similarly, Bairey Merz et al. [29] found that nearly half of American women lack sufficient knowledge about chronic CVDs. These studies reinforce the findings of the present research, highlighting that health education is a crucial need for patients with chronic cardiovascular conditions.

The need for dietary education and nutritional guidance is another significant need for these patients. Mozaffarian [30] emphasizes that understanding nutritional priorities is essential for patients with CVDs, obesity, and diabetes, which aligns with the current study. Schulze et al. [31] also note that knowledge of dietary patterns can help prevent chronic diseases. While this study broadly addresses chronic diseases and dietary patterns, it underscores the importance of nutritional education, consistent with the present findings. Additionally, the need for education on healthy lifestyles is critical. Zhang et al. [32] demonstrate that lifestyle modifications can significantly reduce the risk of chronic CVDs, further highlighting the importance of this educational need.

Taken together, evidence for Health Education was highly consistent across the six contributing studies regardless of design: quantitative studies [16, 21, 26] quantified education as a top‐ranked patient priority, while qualitative studies [14, 17] converged on the same underlying need but articulated it differently, emphasizing the manner of communication individualized, understandable, and dialogic rather than its content. This methodological convergence, summarized in Table 3, indicates strong and consistent evidence for this theme, even though what patients mean by “education” differs depending on how the need was elicited.

Another key need identified in the present study is self‐care management. Jaarsma et al. [33] identify self‐care as a priority in managing HF, which aligns with the current study, although their focus is specifically on HF. The need for symptom monitoring is also crucial. Wang et al. [34] suggest that wearable sensors can monitor cardiovascular health, detect abnormal symptoms, and aid in disease management. While this study focuses on the challenges and benefits of wearable sensors, it supports the importance of symptom monitoring, as highlighted in the present study.

Evidence for Self‐Care Management was likewise consistent across the four contributing studies [14, 15, 19, 22], spanning both survey‐based and interview‐based designs. Notably, the quantitative study by Schäfer‐Keller reported that only a minority of patients achieved adequate self‐care maintenance, management, and confidence, suggesting that although the need for self‐care support is well recognized across the evidence base, patients' actual self‐care capacity frequently falls short of it a gap that a purely descriptive list of themes would otherwise leave invisible.

The need for resources to prevent complications is another critical requirement. Fordyce et al. [35] provide recommendations for preventing complications in CCU settings. Although their study targets healthcare providers, many of their recommendations can also be applied to patients, aligning with the current findings.

The present study also identifies the need for holistic support, which includes emotional, social, spiritual, and financial support. Zhang et al. [32] explore the relationship between socioeconomic status, lifestyle, and mortality from CVDs, finding that better socioeconomic status leads to healthier lifestyles and reduced mortality. These results reinforce the current study's findings. However, Nordin et al. [36] found a weak correlation between emotional support and healthy lifestyle adoption among cardiovascular patients, which contrasts with the present study. This discrepancy may be due to Nordin and colleagues' focus on statistical relationships rather than patient needs.

This tension is worth treating as a substantive finding rather than a discrepancy to note in passing. It suggests that emotional and holistic support needs can be strongly and consistently felt by patients even where they show little measurable association with downstream behavior change, indicating that reported need and demonstrated outcome are not interchangeable forms of evidence, and that qualitative accounts of need should not be discounted simply because they lack corroboration from statistical association studies.

Care coordination is another critical need identified in the present study. Patients require access to healthcare providers even after discharge and benefit from remote care and continuous communication with a care coordinator. Miller et al. [37] found that care coordination in primary care can help manage risk factors for chronic CVDs. Similarly, Murphy et al. [38] suggest that coordinated care can reduce cardiovascular risk factors in patients with mental health disorders. These findings align with the current study.

Evidence for Care Coordination was more limited and more context‐dependent than for the themes above, drawn from only 3 of the 14 studies [13, 16, 23] and concentrated in higher‐income settings (Germany, Spain, Canada). None of the studies conducted in lower‐ or middle‐income settings identified a named care coordinator or telehealth platform as a priority need, emphasizing instead basic access to services and affordability. This asymmetry, expanded on later in this section, suggests that Care Coordination as currently framed in the literature may reflect the priorities and infrastructure of well‐resourced health systems more than a universal patient need.

Given the chronic and progressive nature of many CVDs, patients in advanced stages require end‐of‐life care, including support in decision‐making, palliative care, and spiritual care. Sobanski et al. [39] emphasize that patients with HF in advanced stages need supportive, palliative, and spiritual care. Van Bulck et al. [40] also advocate for reducing aggressive and futile interventions in end‐of‐life stages, focusing instead on palliative care. These findings support the results of the present study.

Evidence for End‐of‐Life Care needs was drawn from four studies [16, 23, 24, 28] and, while directionally consistent, was not uniform in magnitude. DeGroot and colleagues found that physically frail community‐dwelling older adults with HF reported substantially higher unmet palliative care needs than nonfrail patients with the same diagnosis, indicating that frailty may be a stronger determinant of unmet end‐of‐life care need, a distinction the current thematic structure does not yet capture.

While the included studies encompass diverse populations—including patients with HF, hypertension, and women with prior preeclampsia—this heterogeneity is a deliberate feature of this integrative review. By synthesizing findings across these varied cardiovascular conditions, we were able to identify common, universal care needs that transcend specific disease diagnoses, providing a more comprehensive perspective than a review focused on a single condition would allow.

While the five themes identified in this review were consistently reported across the included studies, important variations in the emphasis placed on different care needs were evident. These differences likely reflect variations in healthcare system organization, resource availability, and socioeconomic contexts rather than differences in patients' underlying conditions alone. For example, studies conducted in high‐income countries more frequently emphasized care coordination, telehealth, and digital health solutions, whereas studies from low‐ and middle‐income countries highlighted financial hardship, limited access to healthcare services, and the need for family and community support. These findings suggest that unmet care needs are shaped not only by disease characteristics but also by the capacity of health systems to provide continuous, patient‐centered care [15, 19, 26].

Another important observation is that several care needs remain consistently unmet despite increasing recognition in the literature. This persistent gap may result from fragmented healthcare delivery, limited consultation time, inadequate integration of multidisciplinary services, insufficient health literacy, and the continued predominance of biomedical models that prioritize disease management over psychosocial and supportive care. Consequently, although clinical guidelines increasingly advocate comprehensive and person‐centered care, implementation in routine practice remains inconsistent [13, 14].

Cultural factors also appear to influence the expression and prioritization of care needs. Expectations regarding family involvement, caregiving responsibilities, communication with healthcare professionals, decision‐making, and end‐of‐life care differ substantially across societies. For instance, family members play a central caregiving role in many Asian and African settings, whereas patients in Western healthcare systems may place greater emphasis on autonomy, shared decision‐making, and formal support services. Similarly, spiritual support and discussions surrounding palliative care may be more acceptable and routinely integrative in some cultural contexts than in others [15, 19, 22]. These cultural differences highlight that interventions designed to address care needs should not adopt a universal approach but rather should be adapted to local cultural values, healthcare infrastructure, and available resources.

The results of any study should be considered alongside its limitations. In this study, limitations include limited access to the full text of some articles and the restriction to Persian and English‐language studies due to the researchers' language proficiency. Future research should focus on assessing the cost‐effectiveness of care interventions and conducting comprehensive reviews of studies in languages other than Persian and English.

As an integrative review, this study acknowledges the inherent challenge of synthesizing diverse study designs (quantitative, qualitative, and mixed‐methods). Unlike a systematic review, which focuses on a narrow set of criteria, our integrative approach prioritizes a comprehensive understanding of the complex care needs of patients with chronic CVDs.

## 5 Conclusion

Patients with chronic CVDs have a wide range of needs that must be addressed through the design of specialized care programs. By developing care programs based on the recognized and understood needs of these individuals, we can help them achieve better health and a higher quality of life. Once these needs—often fundamental and vital for their well‐being—are met, it is essential to conduct specialized research using various study methods to identify unmet needs and explore ways to address them. This approach ensures a comprehensive understanding of patient needs and supports the development of effective, patient‐centered care strategies.

## Author Contributions

**Mahdi Nabi Foodani:** conceptualization, writing – original draft, writing – review and editing, validation, methodology, formal analysis, investigation, data curation. **Masoumeh Zakerimoghadam:** conceptualization, methodology, supervision, investigation, writing – original draft, writing – review and editing. **Shahrzad Ghiyasvandian:** writing – original draft, writing – review and editing, conceptualization, validation, data curation, formal analysis. **Verónica V. Márquez Hernández:** conceptualization, investigation, validation, writing – original draft, writing – review and editing, software. **Mª Carmen Rodríguez‐García:** conceptualization, investigation, visualization, supervision, writing – original draft, writing – review and editing. **Amir Hossein Goudarzian:** writing – original draft, writing – review and editing, investigation, formal analysis, data curation. **Zahra Abbasi Dolatabadi:** writing – original draft, writing – review and editing, conceptualization, data curation, supervision, investigation, methodology, project administration. All authors have read and approved the final version of the manuscript.

## Funding

The authors have nothing to report.

## Disclosure

The Zahra Abbasi Dolatabadi affirms that this manuscript is an honest, accurate, and transparent account of the study being reported; that no important aspects of the study have been omitted; and that any discrepancies from the study as planned (and, if relevant, registered) have been explained.

## Ethics Statement

This integrative review compiles data from previously published studies, without the direct involvement of new human or animal subjects or the collection of primary data. Ethical approval and informed consent were secured in the original studies included in this review. The research was conducted following ethical guidelines for reviews, maintaining transparency, accuracy, and compliance with established reporting standards such as PRISMA.

## Consent

The authors have nothing to report.

## Conflicts of Interest

The authors declare no conflicts of interest.

## Data Availability

The data set used in this research is available from the corresponding author upon request. Zahra Abbasi Dolatabadi had full access to all of the data in this study and takes complete responsibility for the integrity of the data and the accuracy of the data analysis.
